# India-Rubber Stoppers for Bottles

**Published:** 1862-01

**Authors:** 


India-Rubber Stoppers for Bottles. *Messrs. Editors*: Will you allow me to call the attention of the profession, through your pages, to a recent invention, which promises to be of great convenience to physicians, as well as to all other classes of the community. I refer to the India-rubber corks for bottles and vials. By means of this admirable substitute for the common cork, bottles containing the strongest acids

---

may be carried without danger in the pocket, and the great inconvenience and expense of ground-glass stoppers obviated. The vulcanized rubber of which they are made will resist, I believe, all chemical agents. I have kept a bottle of concentrated sulphuric ether, closed with one of these stoppers, inverted for several days, without the slightest visible effect being produced on the gum.

For alcohol, solutions of nitrate of silver, tincture of iodine, the various mineral acids, and, indeed, for all liquids, the India-rubber stopper is more than a convenience, it is a positive luxury, as, I am confident, all will agree who have tried it. To practitioners in the country, who carry their own medicines with them, it will be of inestimable advantage. In the sick room, also, the new stopper is of very great utility. By means of it, bottles containing effervescing fluids, such as champagne, citrate of magnesia, soda water, etc., can be tightly corked again after having been opened, so that small quantities of their contents may be used at a time without the slightest detriment to the remainder. Lastly, and not least, these corks are sold at a price which will place them within the reach of all. M.—*Boston Med. & Surg. Journal.*

